# Differential gene expression in aphids following virus acquisition from plants or from an artificial medium

**DOI:** 10.1186/s12864-022-08545-1

**Published:** 2022-04-30

**Authors:** Aurélie Marmonier, Amandine Velt, Claire Villeroy, Camille Rustenholz, Quentin Chesnais, Véronique Brault

**Affiliations:** Université de Strasbourg, Institut National de Recherche en Agriculture, Alimentation et Environnement, SVQV UMR-A1131, 68000 Colmar, France

**Keywords:** *Myzus persicae*, RNA-Seq, Polerovirus, Vector behavior

## Abstract

**Background:**

Poleroviruses, such as turnip yellows virus (TuYV), are plant viruses strictly transmitted by aphids in a persistent and circulative manner. Acquisition of either virus particles or plant material altered by virus infection is expected to induce gene expression deregulation in aphids which may ultimately alter their behavior.

**Results:**

By conducting an RNA-Seq analysis on viruliferous aphids fed either on TuYV-infected plants or on an artificial medium containing purified virus particles, we identified several hundreds of genes deregulated in *Myzus persicae*, despite non-replication of the virus in the vector. Only a few genes linked to receptor activities and/or vesicular transport were common between the two modes of acquisition with, however, a low level of deregulation. Behavioral studies on aphids after virus acquisition showed that *M. persicae* locomotion behavior was affected by feeding on TuYV-infected plants, but not by feeding on the artificial medium containing the purified virus particles. Consistent with this, genes potentially involved in aphid behavior were deregulated in aphids fed on infected plants, but not on the artificial medium.

**Conclusions:**

These data show that TuYV particles acquisition alone is associated with a moderate deregulation of a few genes, while higher gene deregulation is associated with aphid ingestion of phloem from TuYV-infected plants. Our data are also in favor of a major role of infected plant components on aphid behavior.

**Supplementary Information:**

The online version contains supplementary material available at 10.1186/s12864-022-08545-1.

## Background

Most plant viruses are transmitted by phytophagous arthropods, in particular phloem-feeding insects such as aphids [1]. Virus transmission by aphids relies on intricate and complex interactions between aphid and viral components, especially proteins, which define several transmission modes that are classified based on various criteria (e.g. retention times, sites of retention in vectors, virus localization in plant tissues) [2–5]. Physiological and behavioral trait modifications have been associated with virus acquisition by aphids, which also vary depending on the mode of virus transmission [6, 7]. Members of the *Luteovirus* genus (*Tombusviridae* family) and the *Polerovirus* genus (*Solemoviridae* family) formerly in the same *Luteoviridae* family [8] are transmitted by aphids in a circulative and non-replicative manner. The aphid transmission process of these viruses, and the determinants involved have been widely studied.

The aphid-transmitted tombusvirids and solemovirids have a single-stranded RNA genome encapsidated in icosahedral particles and are acquired by aphids during ingestion of phloem sap from infected plants. The virus particles are moved through intestinal and salivary gland cells by transcytosis after being captured on the cell surface by specific receptors [9, 10]. In the hemolymph, viral particles are thought to bind to a bacterial chaperone (referred to as symbionin) that protects them from degradation in the aphid’s body [11]. This mechanism is however controversial because the different localization of viral particles and symbionin is inconsistent with a direct interaction between the two components [12]. During transcytosis within intestinal and salivary gland cells, virus particles are always enclosed within vesicles and are not expected to be in contact with other cellular components except the virus-specific receptors and vesicle membrane components [13–15]. Until now, the understanding of the gene deregulation in aphids following luteovirids acquisition is limited. Deregulation of the expression of genes involved in immunity regulatory-related systems, including lysosome, ubiquitin-mediated proteolysis, insect hormone biosynthesis and proteolysis pathways have been identified in three aphid species *Sitobion avenae*, *Schizaphis graminum* or *Rhopalosiphum padi* after barley yellow dwarf virus (BYDV, *Luteovirus* genus) acquisition from infected wheat [16, 17]. A former transcriptomic analysis on a partial set of intestinal genes deregulated following pea enation mosaic virus (*Enamovirus* genus) uptake by *Acyrthosiphon pisum* revealed a moderate gene deregulation of 1.8% of the genes analyzed, suggesting that the virus particles could hijack a constitutive endocytosis/exocytosis mechanism in aphids [18]. More recently, genes encoding cytochrome p450 or involved in cuticle formation and development were predominantly identified in *Myzus persicae* carrying potato leafroll virus (PLRV, *Polerovirus* genus) [19].

Aphid acquisition of luteovirids from infected plants has been shown to induce modifications of physiological and behavioral parameters in the insects. Aphids carrying PLRV were attracted towards volatiles emitted by non-infected plants [20]. A similar behavior was observed for aphids carrying cucurbit aphid-borne yellows virus (CABYV, *Polerovirus* genus) or BYDV [21, 22]. In these examples, a preference reversal was noticed, also called ‘conditional vector preference’, since non-viruliferous aphids preferred plants infected with PLRV, CABYV or BYDV. Theoretical extrapolations to field scale of these effects in vector preferences after virus uptake predicted a benefit for virus spread [23, 24]. The shift of behavior could result from aphid acquisition of virus particles or plant components altered by virus infection. A direct implication of BYDV particles acquisition on aphid behavior was suggested in the pioneer work by Ingwell et al. [22], who observed a preference reversal after acquisition of purified BYDV particles from an artificial medium. Modification of aphid behavior after virus acquisition may be conducive to virus transmission and has led to the emergence of the ‘host and vector manipulation hypothesis’ [25, 26], which was suggested in many viral pathosystems including the tombusvirid- or solemovirid-aphid combinations (for review [6, 7, 27–29]). Tombusvirids or solemovirids acquisition by aphids from infected plants resulted predominantly in a positive impact on aphid survival, fecundity or both [6]. For example, an increased aphid fitness was observed when aphids fed on BYDV-infected wheat [30]. More recently, it was shown that *R. padi* carrying BYDV exhibited a higher heat tolerance associated with an overexpression of heat-shock protein genes which increased their lifespan and fecundity under heat stress conditions [31]. Similarly, *A.* *pisum* feeding on pea plants infected with bean leafroll virus (*Polerovirus* genus) displayed a higher survival rate and an increased fecundity compared to aphids reared on non-infected plants [32]. Chesnais et al. [33] showed that *M. persicae* carrying turnip yellows virus (TuYV, *Polerovirus* genus) after feeding on infected *Montia perfoliata* displayed an increased locomotor activity, a higher fecundity and an extended capacity to exploit resources by taking less time to reach the phloem, and ingesting more sap. However, these effects are not observed for all aphid-transmitted solemovirids pathosystems, as only minor effects on phloem activities were observed for *Aphis gossypii* carrying CABYV [21]. In conclusion, since similar fitness and behavioral parameters have not been addressed for all specific pathosystems studied so far, it is still unclear which modifications are in common.

We addressed in this work the question of whether gene deregulation in viruliferous aphids and modification of their behavior are related to (i) aphid feeding on infected plants allowing acquisition of infected plant components together with virus particles, or (ii) to acquisition of virus particles only. We used RNA-Seq to analyze gene deregulation in *M.* *persicae* following TuYV acquisition from infected *Arabidopsis thaliana* or from an artificial medium containing purified virus particles. We also performed aphid locomotor behavior assays to analyze the effect of both types of TuYV acquisition on aphids. Our results suggest that acquisition of virus particles is mostly invisible to the cell machinery, since only a few common aphid genes are deregulated at a low amplitude in both acquisition types. Aphids fed on TuYV-infected plants had a locomotor behavior altered, but not those fed on artificial medium containing purified particles. Genes potentially involved in aphid locomotor behavior and signal perception were almost exclusively found in aphids fed on infected plants and not in those acquiring virus particles by feeding on an artificial medium.

## Materiel and methods

### Plant growth and aphid rearing

*Arabidopsis thaliana* Col-0 line (Columbia-0 ecotype) was originally obtained from the NASC germplasm center (University of Nottingham, UK). Plants were grown in a growth chamber for two weeks post-sewing under 20 ± 1 °C and 14 h photoperiod under fluorescent lamps before use. After inoculation by viruliferous or non-viruliferous aphids, plants were kept in the same conditions. The *M.* *persicae* (Sulzer) (Hemiptera: Aphididae) clone was reared on pepper plants (*Capsicum annuum*) in a growth chamber under 20 ± 1 °C, and 16 h photoperiod. Synchronized 8-day old *M.* *persicae* were obtained by transferring parthenogenetic adult females on detached leaves of pepper plants deposited on 1.5% agar in Petri dishes. After 24 h, adult females were removed and first instar (< 24 h-old aphids) were kept on the detached leaves for an additional 7 days.

### Virus inoculation to A. thaliana and TuYV detection by ELISA

*Arabidopsis thaliana* plants were infected with TuYV by agroinfiltration. For this, a pBin plasmid containing the viral sequence was introduced into *Agrobacterium tumefaciens* strain C58C1 [34] and a cell culture was grown to an OD_600_ of 0.5 before being agroinfiltrated into 3-week old *A. thaliana* as reported [35]. Plant infection was assayed by double-antibody sandwich enzyme-linked immunosorbent assay (DAS-ELISA) [36] with a TuYV-specific polyclonal antiserum (Loewe, Sauerlach, Germany). As controls, plants were agroinfiltrated with bacteria carrying an empty pBin plasmid.

### Viruliferous and non-viruliferous aphids for RNA-Seq analysis

Viruliferous 10 day-old *M. persicae* were obtained by feeding 8 day-old aphids for 48 h on TuYV-infected *A. thaliana* (three plants) or on an artificial medium containing 100 ng/µl of virus in sucrose and MP148 (22% sucrose final concentration) [37]. Purified virions were obtained from TuYV-infected *Montia perfoliata* following the procedure described by van den Heuvel et al. [38]. Virus purification from TuYV-infected *M. perfoliata* is highly efficient in contrast to that from *A. thaliana*. The protein contents of the purified suspension prepared from TuYV-infected plants was analyzed by 10% SDS-PAGE after denaturation of the sample for 10 min at 95 °C in Laemmli buffer. The gel was stained with Coomassie blue. A western-blot was performed on the same virus purification extract using coat proteins-specific antisera as described in [39]. Non-viruliferous aphids were obtained by feeding aphids on non-infected *A. thaliana* (two plants) or on an artificial medium containing sucrose and MP148.

After feeding on plants or artificial medium, aphids were collected for RNA extraction. The viruliferous status of the aphids (i.e. their ability to transmit the virus to plants) was controlled in parallel on a sub-fraction of aphids by transferring individual aphids on 2 week-old *A. thaliana* for a 3-day inoculation access period. Aphids were then eliminated with the insecticide Pirimor® (0.5 mg/ml) and test plants were assayed by ELISA 3 weeks after inoculation.

### Aphid RNA isolation

Batches of 30 aphids were collected for each condition and total RNA was extracted using RNeasy minikit (Qiagen, Hilden, Germany) according to the manufacturer’s instructions. For the RNA-Seq analysis, RNA concentration was determined with Qubit (Thermo Fisher Scientific, Waltham, USA) and RNA purity was controlled with a Bioanalyzer (QC Bioanalyser 2100, Agilent Technologies, Santa Clara, USA). For the qRT-PCR analysis, RNA was quantified at 260 nm with the Nanodrop 2000 (Thermo Fisher Scientific, Waltham, WI, USA).

### RNA sequencing and data analysis

RNA sequencing was performed by Fasteris (Plan-les-Ouates, Switzerland) from extracted total RNAs after a mRNA purification step. The 12 cDNA libraries (4 conditions and 3 biological replicates per condition) were generated using TruSeq Stranded mRNA Sample Preparation Protocol, following Illumina’s instructions. Libraries were then sequenced on Illumina Hiseq 3000/4000 sequencer as paired-end 75 base reads following Illumina’s instructions, for a yield of 53.3 GB (from 43.8 M to 66 M reads/sample).

Read quality was first validated using the fastQC tool (v0.11.5). Then, the reads were aligned with STAR aligner (v2.5.3a) on the *M. persicae* clone G006 genome (G006b_scaffolds assembly, https://bipaa.genouest.org/is/aphidbase/myzus_persicae/downloads/), for a total of 91 to 96% of aligned reads. Finally, genes from *M. persicae* Clone G006b scaffolds annotations were quantified with featureCounts tool (v1.5.3), to obtain the 12 count tables used for the differential gene expression analyses. Eighty-three to 88% of reads were assigned to a gene with featureCounts. Two differential gene expression analyses were performed with package R®: one with the DESeq2 package, the reference package for RNA-seq differential expression analysis, using a FDR (False Discovery Rate) ≤ 0.05, and one with the NOISeq package, with an a posteriori probability ≥ 0.95 (equivalent to a FDR ≤ 0.05) to correct a batch effect detected in our data set. In this latter analysis, the batch correction was performed using the ARSyNseq method (ASCA Removal of Systematic Noise for sequencing data) and the differential expression was conducted with the NOISeqBIO method. To interpret the set of genes differentially expressed in the comparisons of interest tested with DESeq2 and NOISeq packages (*M. persicae* on TuYV-infected plants *vs M. persicae* on non-infected plants, and *M. persicae* on medium with purified TuYV *vs M. persicae* on medium without virus) a gene ontology (GO) enrichment analysis was performed with the topGO package of R and the blast2go functional annotations supplied with the G006b assembly (http://bipaa.genouest.org/sp/myzus_persicae/download/annotation/CloneG006_v2/functional_annotation/blast2go.annot). This allows sorting genes in three categories (sub-ontologies) based on their molecular function, biological process and cellular component and provides a representation of their abundance in GO term.

### Quantitative RT-PCR (RT-qPCR)

First-strand cDNA was synthesized from 1 µg of RNA using Moloney murine leukemia virus (MMLV) reverse transcriptase (Promega Corporation, Madison, WI, USA) according to the manufacturer’s instructions, with oligo(dT)18 as the primer. The quantitative PCR (qPCR) reaction was performed using SYBR® Green Supermix (Bio-Rad, Hercules, CA, USA) and specific primers designed by Primer 3 software [40] (Additional file 1). The RT-qPCR analyses were performed in triplicate in 96-well optical plates. Each plate contained the two reference genes samples and the negative control samples together with the target gene samples. Each reaction was performed after mixing 80 ng of cDNA with 0.8 µL of each primer at 10 mM, and 10 µL of SYBR® Green Supermix (Bio-Rad) in a final volume of 20 µL. The RT-qPCR reactions, conducted on a CFX cycler (Bio-Rad), were initiated with a 3 min incubation at 95 °C followed by 40 cycles of amplification (10 s at 95 °C, 30 s at 60 °C). Melt curve analysis was performed from 60 °C to 95 °C with 5 s of 0.5 °C increments. Threshold cycle (CT) values were calculated using Bio-Rad CFX Manager™ software (Bio-Rad). Expression levels were normalized with two aphid reference genes encoding Rpl7 and L27 [41]. The primer specificity was assessed by melting curves analyses of PCR products. Amplification efficiency was controlled by a fivefold dilution series of cDNA (from 1/5 to 1/3125) corresponding to reference and target genes. Both analyses were conducted using Bio-Rad CFX Manager™ software. The relative expression levels were calculated using the 2-∆∆CT method [42]. The samples analyzed by RNA-Seq together with additional aphid samples collected at the same time but not processed by sequencing were included in this analysis. The data of the RT-qPCR and RNA-Seq were analyzed by a Pearson correlation test.

### Aphid locomotor activity

Dispersion behavior and velocity of viruliferous (following virus acquisition from plant or from an artificial medium) and non-viruliferous aphids (deposited on healthy plants or virus-free artificial medium) were monitored on a target arena following the protocol described in Chesnais et al. [33]. Aphid locomotion behavior was recorded by depositing an individual aphid in the center of a paper arena (285 mm diameter), divided into 10 concentric circles (“spatial zone”) spaced by 15 mm. The arena was placed between four white cardboard slabs (45 cm high) to avoid external stimuli. For each aphid, we determined the number of spatial zones crossed (“aphid locomotor or movement activity”), the maximum zone reached (“aphid dispersion”), and the time taken to move from one zone to another for a maximum of 300 s (“aphid velocity”). The test was completed (i) if the aphid crossed the 10^th^ spatial zone and left the arena, or (ii) at the end of the 300 s. Two blocks of 30 aphids were used for each aphid status (viruliferous or non-viruliferous) and each acquisition type (plant or artificial medium).

### Statistical analyses

We used generalized linear models (GLM) with a likelihood ratio and Chi-square test to assess whether there was an effect of the aphid status (viruliferous or non-viruliferous) or of the acquisition type (plant or artificial medium) on *M.* *persicae* locomotor activity. We carried out GLM using a Gamma (link = “inverse”) for time taken to move from one zone to another and GLM using a Poisson (link = “log”) for the number of spatial zones crossed and the maximum zone reached. When a significant effect of one of the main factors was detected or when an interaction between factors was significant, a pairwise comparison using least-squares means (package R: “emmeans”) (*p* value adjustment with Tukey method) at the 0.05 significance level was used to test for differences between treatments. The fit of all GLM was controlled by inspecting residuals and QQ plots.

## Results

### Differential expression of aphid genes after feeding on infected plants

To address the global gene expression in *M. persicae* after feeding on TuYV-infected plants *vs* non-infected plants, RNA-Seq analyses were performed. Before conducting this analysis, the infectious status of the aphids was controlled. After TuYV acquisition on three infected plants, a subset of the potentially viruliferous aphids was transferred onto 30 test plants (one aphid/test plant) and 28 of these plants (93.3%) were infected. All 10 plants inoculated with aphid fed on non-infected plants remained non-infected.

**Fig. 1:**
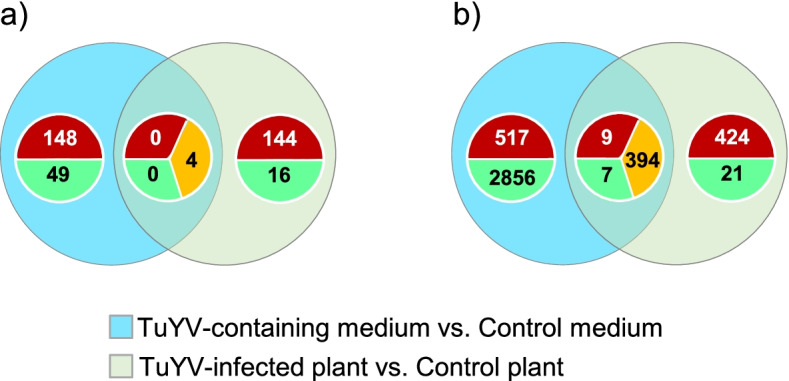
Venn diagrams of differentially expressed genes (DEGs) in *Myzus persicae* fed 48 h on TuYV-infected plants or on an artificial medium containing purified TuYV particles. Number of genes deregulated with an FDR ≤ 0.05. **a** Venn diagram for DESeq2 analysis; **b** Venn diagram for NOISeq analysis. In red: up-regulated or common up-regulated genes; in green: down-regulated or common down-regulated genes; in yellow: contra-regulated genes (up-regulated genes in one group and down-regulated in the other group)

The principal component analysis showed an outlier among the three replicates of *M. persicae* fed on non-infected plant (Plant_Control_2, Additional file 2). This outlier was therefore removed from the analysis. Then, from the 18 529 putative genes of *M. persicae* genome, 164 were identified as differentially expressed genes (DEGs) in the DESeq2 analysis with an FDR ≤ 0.05 (146 DEGs upregulated and 18 DEGs downregulated), when analyzing aphids fed on plants (TuYV-infected *vs* non-infected plants) (Fig. 1a, Additional file 3).

**Fig. 2:**
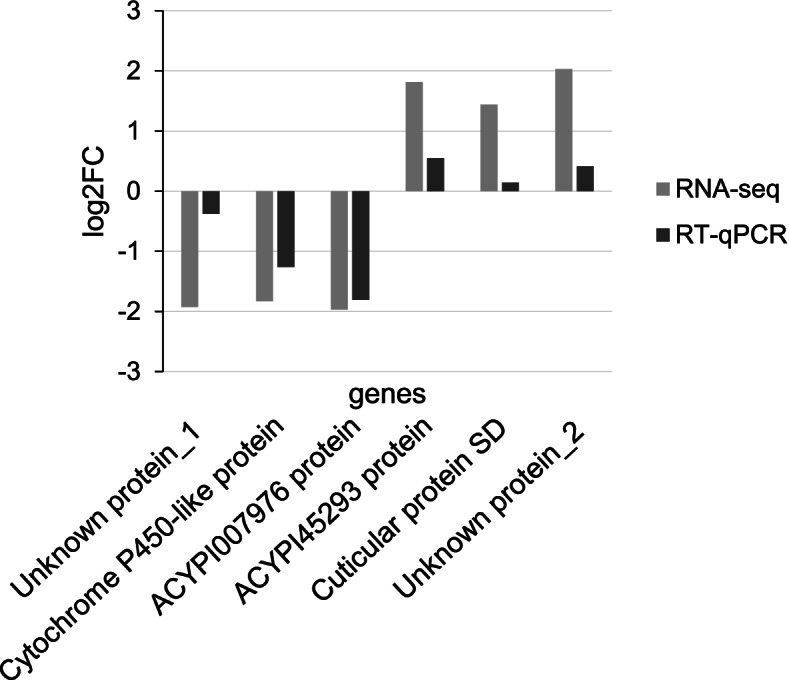
Comparison of gene expression analyzed by RNA-Seq or RT-qPCR. Genes are referred to as their annotation on the *M. persicae* genome. Genes encoding Unknown protein_1, Cytochrome P450-like protein and ACYPI007976 protein were down-regulated genes, and genes encoding the ACYPI45293, Cuticular protein SD and an unknown protein_2 were up-regulated genes in viruliferous vs non-viruliferous aphids. Gene ID, RNA-Seq data, primers used in the RT-qPCR experiments and mean expression of the gene are shown in Additional file 1. Data are presented as the log2 of the ratio: mean expression in viruliferous aphids/mean expression in non-viruliferous aphids

To validate the RNA-Seq results, the expression pattern of the six more deregulated genes (three down-regulated and three up-regulated genes; Additional file 1) were analyzed by RT-qPCR in the samples used for the RNA-Seq analysis (3 samples of aphids from TuYV-infected plants and 2 samples of aphids from non-infected plants). In addition, two viruliferous aphid samples and three non-viruliferous aphid samples, not processed by sequencing, were included in this analysis. The same trend of expression was observed when transcript accumulation was measured by RNA-Seq or by RT-qPCR with a significant statistical correlation (Fig. 2, Additional file 1).

GO terms enrichment analysis was performed with topGO on DEGs identified by the RNA-Seq differential expression analysis using the DESeq2 package. It should be mentioned that among the 18 529 annotated genes on the G006 *M. persicae* assembly, only half (9 967 genes) have a gene ontology annotation, among which 1 371 are encoding “uncharacterized proteins”. This approach identifies major biological processes, molecular functions and cellular components that are affected by TuYV acquisition from infected *A. thaliana* (Additional file 4). DEGs fit in many functional categories, but the percentage of DEGs in each category remained at a low level (Additional file 4). However, the two major cellular components categories of DEGs identified (out of 7) “integral component of membrane” and “intracellular” can be linked to capture and transport of virus particles in aphid cells (Additional file 4c). Similarly, some DEGs fit in biological processes and molecular functions related to virus uptake and intracellular transport (for example, “transmembrane transport”, “receptor activity”, Additional file 4b).

**Fig. 3:**
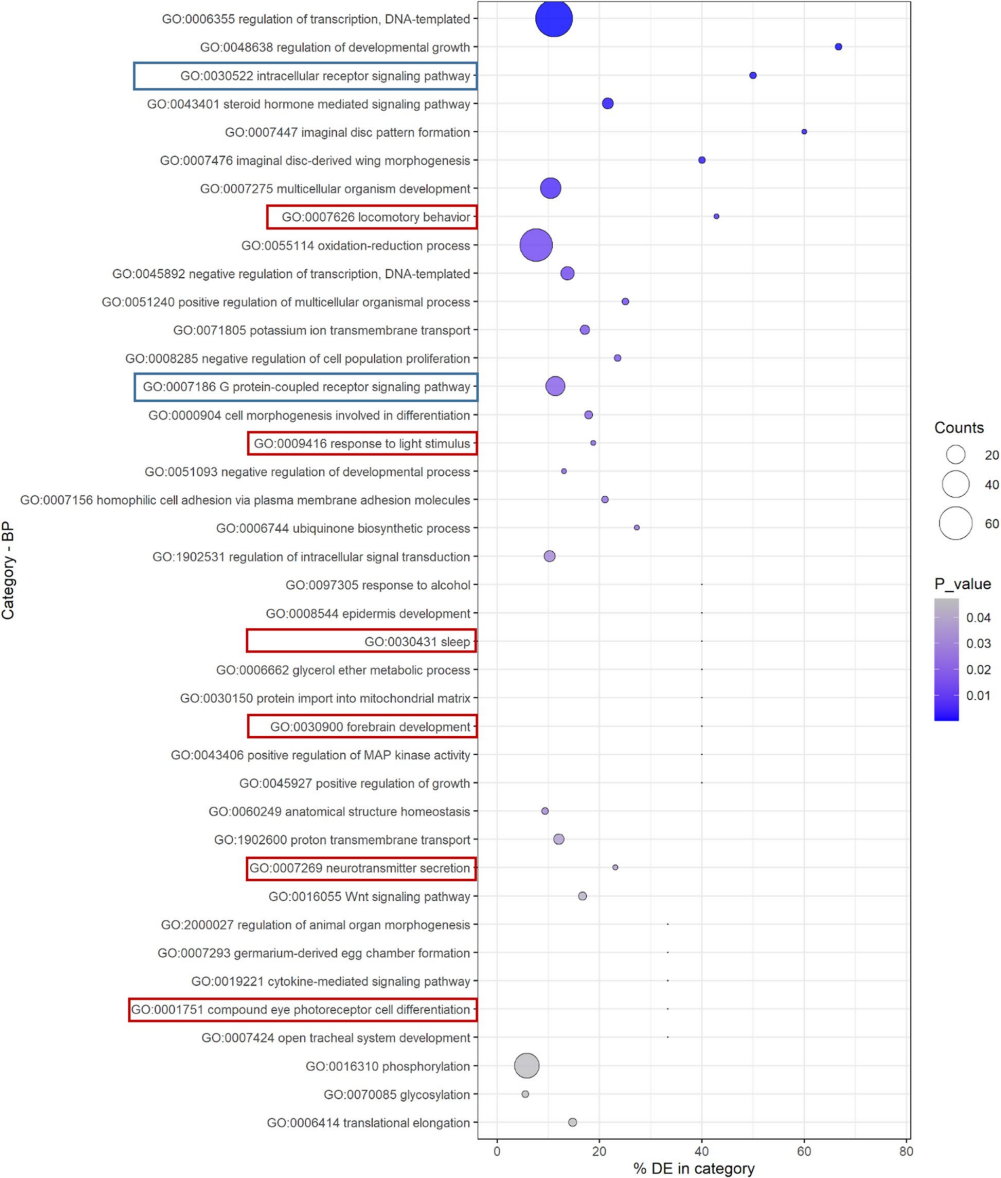
Significant Gene Ontology (GO) categories of biological processes (BP) among the deregulated genes identified by the NOISeq analysis in the aphid *M. persicae* following feeding on TuYV-infected or non-infected plants. The percentage of deregulated genes from the total number of genes included in each GO category is indicated on the horizontal axis (% DE); counts: number of genes differentially expressed in the GO term. GO term boxed in blue represents categories potentially implicated in virus uptake and intracellular transport and GO term boxed in red categories potentially involved in aphid behavior and signal perception

Considering the batch effect shown by the PCA (Additional file 2a) that reduces the power of the statistical analyses and the number of DEGs, another differential expression analysis was conducted using NOISeq. This non-parametrical method allows a batch correction on our data (Additional file 5) and was more efficient in identifying low expression deregulations that were undetected using DESeq2. Four out of the six genes selected after the DESeq2 analysis and processed by RT-qPCR (Fig. 2) were still statistically affected after NOISeq analysis. All the 12 samples were included in this analysis. Using this method, 855 differentially regulated genes were found after virus acquisition from plants (with an a posteriori probability ≥ 0.95) (Fig. 1b, Additional file 6). Five cellular components categories were attributed to the 855 DEGs in aphids fed on plant and four of them (“cytoskeleton”, “plasma membrane”, “heterotrimeric G-protein complex” and “integral component of membrane”, Additional file 7b) could be related to virus uptake and transport in aphid cells. In addition, DEGs in the biological processes and molecular functions such as “intracellular receptor signaling pathway”, “G-protein-coupled receptor signaling pathway” and “structural constituent of the cuticle” could also be implicated in receptor-mediated endocytosis (Fig. 3; Additional file 7a). Interestingly, several genes fit in biological process categories potentially involved in aphid locomotion, and signal perception/transmission, like the categories “locomotor behavior”, “response to light stimulus”, “sleep”, “forebrain development”, “neurotransmitter secretion” and “compound eye photoreceptor cell differentiation” (Fig. 3, Table 1).

**Table 1:** Differentially expressed genes (DEGs) in *M.* *persicae* fed on TuYV-infected plants fitting in GO categories potentially involved in behaviour and signal perception and transmission

**GO category**	**Gene ID** **MYZPE13164_G006_v1.0**	**Annotation**	**Log2FC (NOISeq)**	**Prob (NOISeq)**^a^
GO:0007626 locomotory behavior	_000096010 _000127320 _000138670	-btb poz domain-containing protein kctd16 -protein deadpan -potassium sodium hyperpolarization-activated cyclic nucleotide-gated channel 2 isoform	0.62 0.38 0.23	0.99 0.97 0.97
GO:0009416 response to light stimulus	_000138470 _000138670 _000195310	-calmodulin -potassium sodium hyperpolarization-activated cyclic nucleotide-gated channel 2 isoform -ras-like protein 1	0.22 0.23 0.16	0.99 0.98 1.00
GO:0030431 sleep	_000096010 _000138670	-btb poz domain-containing protein kctd16 -potassium sodium hyperpolarization-activated cyclic nucleotide-gated channel 2 isoform	0.63 0.23	0.99 0.97
GO:0030900 forebrain development	_000143970 _000195310	-homeobox protein engrailed-1a-like -ras-like protein 1	0.44 0.16	0.95 1.00
GO:0007269 neurotransmitter secretion	_000003100 _000138670 _000144190	-synapsin -potassium sodium hyperpolarization-activated cyclic nucleotide-gated channel 2 isoform -ap-2 complex subunit mu	0.13 0.23 0.24	0.96 0.98 0.96
GO:0001751 compound eye photoreceptor cell differentiation	_000138470 _000144830	-calmodulin -ubiquitin-conjugating enzyme e2-17 kda	0.22 0.19	0.99 0.99

^a^Probability of differential expression. Significant at more than 95% when Prob ≥ 0.95

### Differential expression of aphid genes after virus acquisition from an artificial medium

Before conducting the RNA-Seq analysis, the protein content of the purified virus used for aphid acquisition was analyzed by SDS-PAGE followed by Coomassie staining (Additional file 8). In addition to the major coat protein of 22 kDa and the minor coat protein of about 55 kDa, an additional non-viral major band of about 90 kDa was present in the purified viral preparation. This protein was already observed in purified preparations of TuYV [43]. This plant protein, together with possible trace proteins undetected by Coomassie blue staining, is also potentially acquired by the aphids during membrane feeding. The viruliferous status of the aphids was controlled by transferring individual aphids fed on an artificial medium containing purified virus on test plants. All 20 aphid-inoculated plants were infected and the 10 control plants (plants inoculated with aphids fed on artificial medium only) remained non-infected.

RNA-Seq differential expression analysis using the DESeq2 package identified 201 DEGs (150 DEGs upregulated and 51 DEGs down-regulated) when comparing the transcriptome of aphids fed on an artificial medium containing or not purified TuYV particles (Fig. 1a, Additional file 3). No specific GO enrichments was observed in this condition using DESeq2 package (Additional file 9) but we noticed that gene deregulations affected a broader diversity of cellular components (17 categories) when compared to the plant feeding condition (Additional files 9c and 4c). The two predominant categories (“intracellular membrane-bounded organelle” and “intracellular”) may be linked to intracellular transport of viral particles (Additional file 9c). In the molecular function category, the “transmembrane transporter activity” could be relevant for a receptor-based mechanism resulting in virus acquisition (Additional file 9b). No DEGs fitting in a biological process linked to virus uptake and transport or aphid behavior was observed in this condition (Additional file 9a).

When applying the NOISeq analysis on these RNA-Seq data, 3 783 deregulated genes were found following virus acquisition from an artificial medium. The number of DEGs in the aphids fed on artificial medium was higher compared to DEGs in aphids fed plants (Fig. 1b). The 3 783 DEGs fit in 27 different cellular component categories (Additional file 10c), among which the “membrane protein complex”, the “Arp2/3 protein complex” and the “vesicle tethering complex” and “intracellular” could be related to virus particles transcytosis process. Contrary to DEGs in viruliferous aphids fed on plants, no DEGs in aphids fed on the artificial medium could be attributed with confidence to a biological process related to locomotion or signal perception (Additional file 10a, b & c).

### Common deregulated genes after virus acquisition from plant or from an artificial medium

Surprisingly, only four DEGs were in common in aphids fed on infected plants and in aphids fed on the artificial medium containing purified TuYV when the DESeq2 analysis was applied to the RNA-Seq data, but the deregulation of each gene was in opposite direction for the two conditions (Fig. 1a, Additional file 11). With NOISeq analysis, the number of common deregulated genes reached 410 (Fig. 1b) but as already observed with the DESeq2 analysis, most of the common genes were deregulated in opposite directions (Fig. 1b), except 16 genes which followed a similar trend of regulation (Table 2). The deregulations affecting the 16 genes were, however, of rather low amplitude ranging from 1.11 (Log2FC -0.15) to 1.67 (Log2FC -0.74) fold down-expression to 1.05 (Log2FC 0.07) to 1.38 (Log2FC 0.46) fold over-expression.

**Table 2:** Sixteen common genes differentially expressed in viruliferous aphids after virus acquisition from plants or from an artificial medium

^**a**^**Gene ID** **MYZPE13164_G006_v1.0**	**Annotation**	^**b**^**Log2FC on artificial medium**	^c^**Prob. on artificial medium**	^**d**^**Log2FC on plants**	^c^**Prob. on plants**
_000076360	Unknown protein	-0.49	0.992	-0.74	1
_000118000	ACYPI006605 protein	-0.47	0.993	-0.27	0.951
_000124570	ACYPI007976 protein	-0.47	0.960	-0.42	0.951
_000063440	Nitrilase and fragile histidine triad fusion protein NitFhit-like Protein	-0.28	0.989	-0.25	0.955
_000131110	Chitin deacetylase 2B	-0.21	0.960	-0.15	0.999
_000013770	Fatty acyl-CoA reductase 1	-0.19	0.984	-0.18	0.958
_000090420	Tubulin beta-1 chain	-0.18	0.993	-0.19	0.999
_000122920	ADP/ATP translocase	0.12	0.994	0.07	0.972
_000128850	Mitogen-activated protein kinase kinase kinase	0.16	0.979	0.12	0.952
_000145700	Lethal(2)essential for life protein-like protein	0.16	1	0.10	0.987
_000051530	Cysteine and histidine-rich protein 1 like protein	0.18	0.981	0.17	0.983
_000168970	Unknown protein	0.23	0.958	0.14	0.972
_000022550	BCL2/adenovirus E1B 19 kDa protein- interacting protein 3-like protein	0.24	1	0.15	0.965
_000104270	Cathepsin B	0.32	0.981	0.37	0.999
_000002070	Unknown protein	0.39	0.975	0.38	0.976
_000043170	ACYPI50089 protein	0.46	0.999	0.21	0.962

^a^DEGs have been identified by the NOISeq analysis

^b^Log2FC of DEGs in aphids after TuYV acquisition from an artificial medium

^c^probability of differential expression. Significant at more than 95% when Prob ≥ 0.95

^d^Log2FC of DEGs in aphids after TuYV acquisition from plants

### Effect of TuYV acquisition from infected plants or artificial medium on M. persicae behavior

**Fig. 4:**
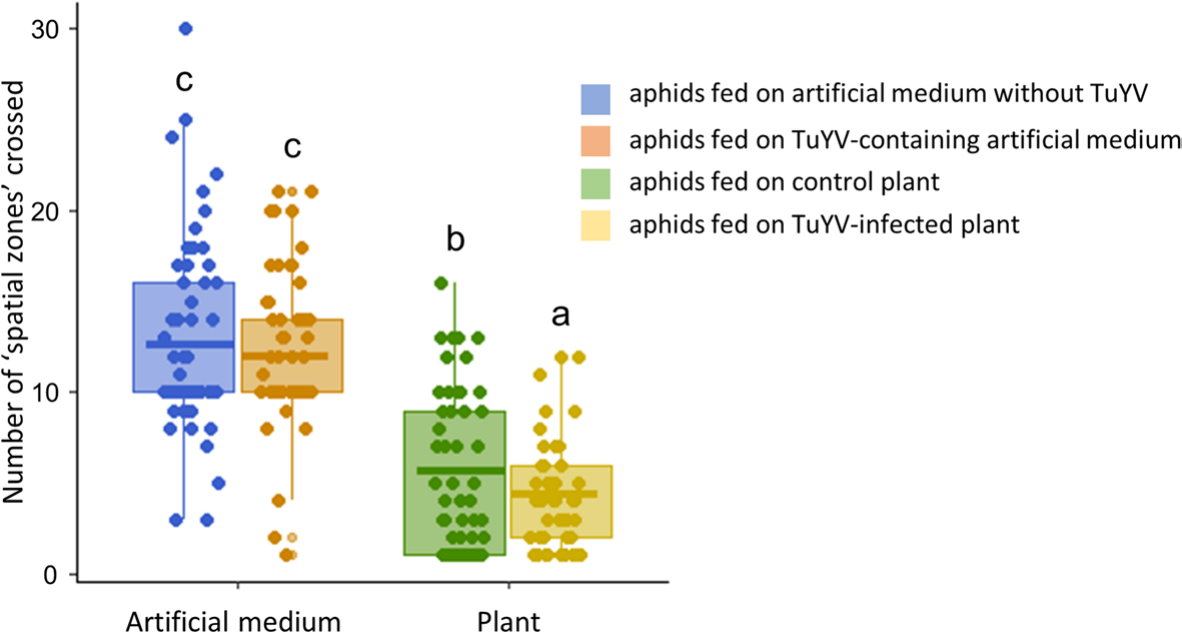
Locomotor activity (number of spatial zones crossed) of viruliferous and non-viruliferous *M. persicae* fed on plants or on artificial medium. Box plots show median (line), 25–75% percentiles (box) and 10–90% percentiles (whisker). Letters indicate significant differences between aphid status with the GLM followed by multiple comparisons; *p*-value < 0.05

We addressed whether TuYV acquisition by *M.* *persicae* from infected plants or from an artificial medium affected the locomotor activity of viruliferous aphids when compared to non-viruliferous aphids. No significant differences for the three locomotor parameters measured were observed between viruliferous and non-viruliferous aphids fed on artificial medium (Fig. 4 and Additional file 12a & b). However, aphids fed on infected plants displayed a significantly lower locomotor activity compared to non-viruliferous aphids (estimated marginal mean pairwise comparisons, *p*-value = 0.025) as shown by the reduced number of spatial zones crossed by viruliferous aphids (Fig. 4). It is worth noting that, when compared to aphids fed on plants, we observed a higher mobility of aphids fed on the artificial medium with a higher number of spatial zones crossed, a farther zone reached and a reduced time spent in each zone. These effects were, however, not linked to the presence of the TuYV in aphids, since they were observed for both viruliferous and non-viruliferous aphids (Fig. 4 and Additional file 12 a & b).

## Discussion

Efficient acquisition of circulative viruses requires vector-host compatibility to ensure a sustained settling and feeding behavior together with an extended phloem sap ingestion phase. It also relies on virus-vector compatibility to promote virus uptake and internalization into vector cells driven by specific receptors. The successive steps of endocytosis and exocytosis to enter and exit vector cells may induce gene deregulations, which could potentially alter aphid behavior. In this paper, we addressed the question of whether the gene deregulations and possible modification of the behavior of viruliferous aphids are related to the ingestion of plant material altered by virus infection or to the acquisition of virus purified particles.

Hundreds of DEGs were detected in viruliferous aphids fed on TuYV-infected plants, whatever the methods used to identify the deregulations (DESeq2 and NOISeq). The maximum changes in gene expression ranged from 39.4-fold down-regulation to 4.6-fold up-regulation for the DESeq2 analysis, and from 4.6-fold down-regulation to 2.8-fold up-regulation for the NOISeq analysis. However, the mean changes in gene expression were low and did not exceed 3.03-fold down-regulation and 1.93-fold up-regulation, for the DESeq2 analysis, and 1.29-fold down-regulation and 1.27-fold up-regulation, for the NOISeq analysis.

### Common differentially expressed genes potentially involved in virus uptake and transport in aphid cells

Sixteen DEGs were common to both types of virus acquisition (from the plant or from the medium). The amplitude of the common genes deregulation was very low and did not exceed a 1.4- and 1.7-fold increase or reduction, respectively. However, these DEGs, in response to acquisition of virus particles from plant or artificial medium, must be attributed to direct effects of virus uptake and transcytosis alone because the virus does not replicate in insect cells.

Of the sixteen DEGs common to both types of acquisition, seven were down-regulated genes. These included a gene encoding a protein implicated in the semaphorin-plexin signaling pathway (MYZPE13164_G006_v1.0_000118000). One function of semaphorins is alteration of the cytoskeleton and therefore potentially modification of the vesicular transport of virions. It is worth noting that among the common down-deregulated genes was *tubulin β1 chain* gene encoding a structural constituent of the cytoskeleton. Semaphorins can be transmembrane proteins acting as receptors [44], but no virus receptor function of semaphorins has been reported. Semaphorins are known to be associated with the development of the nervous system of insects [45]. The outcome of this pathway also involves MAP kinases [46] and one gene in this family was found to be overexpressed in our analysis (*mitogen-activated protein kinase kinase kinase*, Table 1). Whether the MAPK kinase identified here is specifically involved in the semaphoring-plexin pathway would require further analysis.

Two genes slightly down-regulated in viruliferous aphids regardless of the mode of acquisition (*chitin deacetylase-like 4* and *fatty acyl-CoA reductase 1*) are linked to chitin synthesis or modification. Chitin is the major structural component of the exoskeleton of arthropods and a component of the peritrophic matrices in insects. Chitin deacetylases are chitin degrading enzymes leading to the synthesis of chitosan. Their activity can have an impact on insect growth and development [47]. TuYV acquisition by aphids was indeed reported to reduce aphid growth [48]. In addition to their implication in lipid metabolism [49], fatty acyl-CoA reductases are involved in the synthesis of cuticular hydrocarbons of insects [50]. Although these particular two proteins have never been reported to affect virus transmission by insects, other proteins related to chitin are involved in plant virus transmission. Cuticular proteins play a role in non-circulative virus transmission [51]. Implication of cuticular proteins in circulative plant virus transmission is more hypothetical, but these proteins have been identified in several screens looking for virus-associated proteins in aphids [52, 53]. In addition, many DEGs in aphids carrying PLRV were related to cuticle formation [19].

The protein “lethal(2) essential for life protein” belongs to the small heat shock protein family and its expression was slightly increased in viruliferous aphids. Upregulation of heat shock proteins of the HSP70 family has already been observed in aphids carrying BYDV [31]. This deregulation was associated with thermal tolerance of aphids. The sensitivity of aphids carrying TuYV to increased temperatures could be addressed in future projects.

The “BCL2/adenovirus E1B 19 kDa protein-interacting protein 3-like protein” (Bnip3L) is known, in virus-infected human cells, to inhibit apoptosis, a cellular defense mechanism against viruses [54]. In humans, this protein localizes at the outer membrane of mitochondria when overexpressed [55]. This gene was moderately over-expressed in viruliferous aphids and whether this human orthologous protein plays a similar function in insect cells is still unknown. Apoptosis is not always antiviral and can have a positive role on virus dissemination in insects, as illustrated by mosquitoes transmission of arboviruses where disruption of the basal lamina of the midgut epithelium induced by apoptosis allows togaviruses to access more rapidly the hemolymph [56]. Apoptosis has also been observed in mosquitoes’ salivary glands after arboviruses infection [56]. TuYV could be perceived as a pathogen and apoptosis could be a defense mechanism of aphid cells after virus endocytosis, although cytopathic effect of TuYV endocytosis has never been observed by transmission electron microscopy [13].

CathepsinB (CathB), whose expression was slightly increased after TuYV uptake, may be one of the most relevant candidates with regard to virus transmission. CathB is an aphid gut cysteine protease whose expression depends on the host-plant used to rear aphids [57]. Inhibition of CathB expression has a beneficial effect on PLRV (another polerovirus) transmission by aphids. The increased activity of CathB observed in aphids reared on turnip could degrade phloem protein potentially protecting virions or facilitating virus recognition and uptake at the gut level [58]. Regulation of Cath expression was supposed to relate on components present in the sap that would be ingested together with the virus. This hypothesis now needs to be reconsidered in the light of our results, since CathB overexpression is also observed after feeding on purified virus suspension, which is mostly deprived of other proteins except virus structural proteins (Additional file 2). The over-expression of this gene could potentially reduce transmission of TuYV if we consider that cathepsins have a common function in polerovirus transmission by aphids.

### Factors responsible for unique differentially expressed genes in aphids after feeding on infected plants or on an artificial medium containing virus particles

Besides the sixteen DEGs in common, we found genes that were differently deregulated depending on whether virus uptake was from infected plants or from artificial medium. The unique gene deregulations in aphids fed on infected plants were of low amplitude and could be attributed to the ingestion of plant material altered by TuYV-infection and acquired during probing in epidermal or mesophyll cells, or during phloem sap ingestion. Secondary metabolites (e.g. the insect deterrent glucosinolates found in *Brassicaceae*), phytohormones, proteins, mRNA, small RNA or miRNAs present in sap and potentially acquired by aphids together with the virus particles could be responsible for gene deregulations [59–61]. Alternatively or additionally, a modification of the sap content in primary metabolites, e.g. the ratio of sugars and amino acids, whose concentration in phloem sap is interdependent [62], could also be responsible for aphid gene expression deregulation. Although no modification of the amino acid composition of the sap collected from TuYV-infected *A. thaliana* was observed, a reduced accumulation of sugars was recorded which affects the sugars/amino acids ratio in the sap ingested by aphids (data not shown).

A high number of unique DEGs occurred following purified virus acquisition but the amplitude of their deregulations was low. It is less likely that these DEGs are induced by virus particles only but rather induced by the ingestion of plant proteins present in the purified suspension from homogenized plants and concentrated compared to their biological concentrations in phloem, if there are ever present there. This would explain their unique occurrence in aphids feeding on TuYV-containing artificial medium. Indeed, any components acquired together with the virus particles may potentially affect gene expression greater than acquisition of the virus particles alone, presenting a methodological challenge for detecting DEGs uniquely in response to virus acquisition alone.

### Behavior modification of aphids after feeding on infected plants

It has been shown that virus-induced modifications of host phenotypes can alter vector-plant interactions, and therefore virus transmission [7, 27]. More recently, it has been suggested that virus acquisition can also modify aphid behavior [20–22]. Interestingly, we observed that TuYV acquisition from infected *A. thaliana* decreased aphid locomotor activity while virus acquisition from an artificial medium had no impact. The reduced locomotor activity of viruliferous aphids fed on TuYV-infected *A. thaliana* is in contrast with results obtained by Chesnais et al. [33], who observed an increased aphid locomotor activity after TuYV acquisition on *M. perfoliata*. These contrasting effects may be due to the plant species used to feed aphids and therefore to plant components acquired together with the virus particles. The absence of aphid behavior modifications after acquisition of virus particles from an artificial medium is also in contrast to a previous study from Ingwell et al. [22] which showed that in vitro acquisition of purified BYDV particles by *R. padi* resulted in an aphid preference for non-infected wheat plants. The discrepancies between the reports may be a consequence of the nature of the pathosystem used in these studies (different virus species, different plant species, different aphid species) or to the specifics of the bioassays performed.

### Unique differentially expressed genes in aphids fed on plants potentially involved in aphid behavior

Several DEGs unique to aphids acquiring the virus from infected plants fit in the behavior and signal perception categories, although it should be mentioned that the amplitude of the deregulation of the genes indicated thereafter is very low (Table 1). Among these, the *btb pox domain-containing protein kctd16* gene is a good candidate to explain aphid behavior modification. KCTD16 protein is an auxiliary subunit of GABAB receptors and its modification has been shown to affect behavioral response of mice [63]. In addition, a role of GABAB receptor in the mice circadian organization of sleep has been reported [64]. We also identified another candidate gene, potentially implicated in the rhythmic behavior of the insects, the *ras-like protein 1* gene, which is involved in signaling in the circadian clock cells in *Drosophila melanogaster* [65]. Finally, two other genes could be potentially involved in the locomotion behavior of insects and responsible for the altered locomotor activity observed for TuYV-viruliferous aphids: (i) the *protein deadpan* gene, deregulated in viruliferous aphids, and for which, a loss of function in *D. melanogaster* resulted in weak motor activities and lethargic behavior [66]; (ii) the *homeobox protein engrailed-1a-like* gene, which has motor function in mice [67] and could control locomotor activity of aphids.

A calmodulin-encoding gene was also deregulated in aphids acquiring the virus by feeding on plants. Calmodulin was shown to affect olfactory performance of *D.* *melanogaster* and odor-guided behavior by impacting the ability of odorant receptors containing a calmodulin-binding site to detect volatile cues [68].

The *synapsin* gene is a candidate to explain behavioral modifications of viruliferous aphids. Synapsins are phosphoproteins which modulate neurotransmitter release. A role of synapsin in *D. melanogaster* development and olfactory-related behavior was described before [69]. Also, a role in neurotransmission and behavior of *hyperpolarization-activated cyclic nucleotide-gated channels 2* gene, which is deregulated in viruliferous aphids, may be suspected, based on its function in mice or in *D. melanogaster* where disruption leads to a range of behavioral defects [70, 71]. Another deregulated gene found in the categories of genes potentially involved in aphid behavior is the *ap-2 complex subunit* gene. Deregulation of this gene may impair synaptic transmission, as shown in drosophila, and potentially insect behavior, but this clathrin-associated adaptor protein could also play a role in virus endocytosis [72].

Another promising candidate among DEGs uniquely occurring in aphids fed on infected plants is the gene encoding an E2 ubiquitin-conjugating enzyme of 17 kDa, which is implicated in eye development of *D. melanogaster* and potentially involved in photoreception [73]. It is conceivable that this or similar genes could be involved in altered plant perception by viruliferous aphids [20–22].

To analyze the function of all DEGs hypothetically affecting aphid behavior, RNA interference technologies could be used [10, 74–77].

## Conclusions

The data presented in this paper compared gene differential expression following virus acquisition by aphids from plant or from an artificial medium. The few common genes between the two modes of virus acquisition are deregulated at a low level and are potentially involved in virus uptake and transcytosis, but also in cellular functions such as apoptosis, chitin synthesis and stress response. These genes potentially represent genes deregulated uniquely by the virus particles. The unique genes deregulated in aphids after virus acquisition from the artificial medium likely represent aphid responses to plant contaminants present in the purified virus suspension. Some of the unique genes deregulated following virus acquisition from infected plants could also be linked to virus transcytosis, but also, to aphid behavior and perception. Compared to non-viruliferous aphids, aphids fed on TuYV-infected plants showed an altered behavior in contrast to aphids fed on an artificial medium. Our results reinforce the general hypothesis that acquisition of plant material altered by virus infection has a greater impact on aphid behavior than acquisition of virus particles per se.

## Supplementary Information

**Additional file 1.** Comparison of the expression of 6 genes by RNA-Seq and RT-qPCR analyses.

**Additional file 2.** Principal component analysis (PCA) of the RNA-seq data showing samples distribution of DESeq2 analysis before (a) and (b) after removal of Plant_Control_2 sample. See Additional file 3 for raw data.

**Additional file 3.** List of DEGs with the DESeq2 analysis (Plant_with_TuYV vs Plant_Control and Medium_with_TuYV vs Medium_Control).

**Additional file 4.** Significant Gene Ontology (GO) categories of (a) biological process (BP), (b) molecular function (MF), (c) cellular components (CC) among the deregulated genes identified by the DESeq2 analysis in the aphid *M. persicae* following feeding on TuYV-infected or non-infected plants. The percentage of deregulated genes from the total number of genes included in each GO category is indicated on the horizontal axis (% DE); counts: number of genes differentially expressed in the GO term. GO term boxed in blue represents categories potentially implicated in virus uptake and intracellular transport and GO term boxed in red categories potentially involved in aphid behavior and signal perception.

**Additional file 5.** Principal component analysis (PCA) of the RNA-Seq data showing samples distribution (NOISeq analysis) before (a) and (b) after batch correction. See Additional file 6 for raw data.

**Additional file 6.** List of DEGs with the NOISeq analysis (Plant_with_TuYV vs Plant Control and Medium_with_TuYV vs Medium_Control).

**Additional file 7.** Significant Gene Ontology (GO) categories of (a) molecular function (MF), (b) cellular components (CC) among the deregulated genes identified by the NOISeq analysis in the aphid *M. persicae* following feeding on TuYV-infected or non-infected plants. The percentage of deregulated genes from the total number of genes included in each GO category is indicated on the horizontal axis (% DE); counts: number of genes differentially expressed in the GO term. GO term boxed in blue represents categories potentially implicated in virus uptake and intracellular transport and GO term boxed in red categories potentially involved in aphid behavior and signal perception.

**Additional file 8.** Protein content of the purified extract prepared from TuYV-infected *M. perfoliata* and used to feed aphids artificially before RNASeq analysis. (a) Polyacrylamide gel electrophoresis of the TuYV purified extract (3 µg) stained with Coomassie blue; (b) Western blot analysis of the same purified extract. The blot was incubated with a mixture of antisera specific for the CP and RT*. CP: major coat protein (CP); RT*: minor coat protein (RT*). The band corresponding to a major plant protein (P90) in the TuYV purified preparation is indicated. The molecular mass markers are indicated in kDa.

**Additional file 9.** Significant Gene Ontology (GO) categories of (a) biological process (BP), (b) molecular function (MF), (c) cellular components (CC) among the deregulated genes identified by the DESeq2 analysis in the aphid *M. persicae* following feeding on artificial medium containing TuYV purified particles or artificial medium only. The percentage of deregulated genes from the total number of genes included in each GO category is indicated on the horizontal axis (% DE); counts: number of genes differentially expressed in the GO term. GO term boxed in blue represents categories potentially implicated in virus uptake and intracellular transport.

**Additional file 10.** Significant Gene Ontology (GO) categories of (a) biological process (BP), (b) molecular function (MF), (c) cellular components (CC) among the deregulated genes identified by the NOISeq analysis in the aphid *M. persicae* following feeding on artificial medium containing TuYV purified particles or artificial medium only. The percentage of deregulated genes from the total number of genes included in each GO category is indicated on the horizontal axis (% DE); counts: number of genes differentially expressed in the GO term. GO term boxed in blue represents categories potentially implicated in virus uptake and intracellular transport.

**Additional file 11.** Common deregulated genes in aphids between plant and artificial medium feeding conditions with the DESeq2 program.

**Additional file 12.** Locomotor activity of viruliferous and non-viruliferous *M. persicae* fed on plants or on artificial medium. (a) maximum zone reached and (b) duration (in seconds) spent in each zone. Box plot show median (line), 25-75% percentiles (box) and 10-90% percentiles (whisker). Letters indicate significant differences between aphid status with the GLM followed by multiple comparisons; *p*-value<0,05.

## Data Availability

The datasets supporting the conclusions of this article are available on the ENA with the following accession number PRJEB46814 (rawdata available for the 12 RNA-seq samples). (Direct link: https://www.ebi.ac.uk/ena/browser/view/PRJEB46814?show=reads).
