# Supplementary material for: Differential gene expression in aphids following virus acquisition from plants or from an artificial medium
Source: BMC Genomics. 2022 Apr 30;23:333. doi: 10.1186/s12864-022-08545-1 (PMC9055738; doi:10.1186/s12864-022-08545-1)
Supplement: Supplementary file 1 — Additional file 1. Comparison of the expression of 6 genes by RNA-Seq and RT-qPCR analyses. [file 12864_2022_8545_MOESM1_ESM.pdf]

Additional file 1 : comparison of the expression of 6 genes by RNA-Seq and RT-qPCR analyses

Gene expression measured by RNA-Seq	Gene ID	Name	Annotation	Log2FC	Adjusted p-value	Relative expression in non-viruliferous aphids[a] by RT-qPCR	Relative expression in viruliferous aphids[a] by RT-qPCR	Log2FC by RT-qPCR	Primer sequences used for RT-qPCR
**Down-regulated genes**	MYZPE13164_G006_v1.0_000085220	A	Unknown protein	-1,93	9,78E-06	0,85 ± 0,10	0,65 ± 0,11	-0,39	5’-CCAACCAACTGATGGTGAGTACAT-3’<br>5’-CTATTTGGGCTATGGCATTTTTGAG-3’
	MYZPE13164_G006_v1.0_000173830	B	Cytochrome P450-like protein	-1,83	9,78E-06	0,65 ± 0,16	0,27 ± 0,09	-1,26	5’-GCCAAGTACCCGTGGTTTGGA-3’<br>5’-TGCATCACACGCGAAAACGAC-3’
	MYZPE13164_G006_v1.0_000141690	C	ACYPI007976 protein	-1,97	7,20E-04	0,59 ± 0,19	0,17 ± 0,01	-1,81	5’-ATGGCCGATGTCGGCGTTAG-3’<br>5’-CTTGCACTTTGCAGTCGCCG-3’
**Up-regulated genes**	MYZPE13164_G006_v1.0_000070160	D	ACYPI45293 protein	+1,81	2,10E-23	0,59 ± 0,08	0,86 ± 0,09	+0,55	5’-GTAACGCTGTTGTTGGCCGC-3’<br>5’-GACCAAAGCTGGAGCAGGGG-3’
	MYZPE13164_G006_v1.0_000097260	E	Cuticular protein SD	+1,44	6,50E-04	0,41 ± 0,10	0,46 ± 0,17	+0,14	5’-TGTCGATCGGTAGAGGTCGTGT-3’<br>5’-ACGTCTTCATCGGCAATGCT-3’
	MYZPE13164_G006_v1.0_000057020	F	Unknown protein	+2,03	2,02E-03	0,61 ± 0,05	0,81 ± 0,09	+0,41	5’-TCAATCGTCAGCGGAGTGCG-3’<br>5’-TCGGAAACGACAACGCCAGG-3’

[a]relative expression mean of 5 samples ± standard deviation
