# Supplementary material for: Differential gene expression in aphids following virus acquisition from plants or from an artificial medium
Source: BMC Genomics. 2022 Apr 30;23:333. doi: 10.1186/s12864-022-08545-1 (PMC9055738; doi:10.1186/s12864-022-08545-1)
Supplement: Supplementary file 5 — Additional file 5. Principal component analysis (PCA) of the RNA-Seq data showing samples distribution (NOISeq analysis) before (a) and (b) after batch correction. See Additional file 6 for raw data. [file 12864_2022_8545_MOESM5_ESM.pdf]

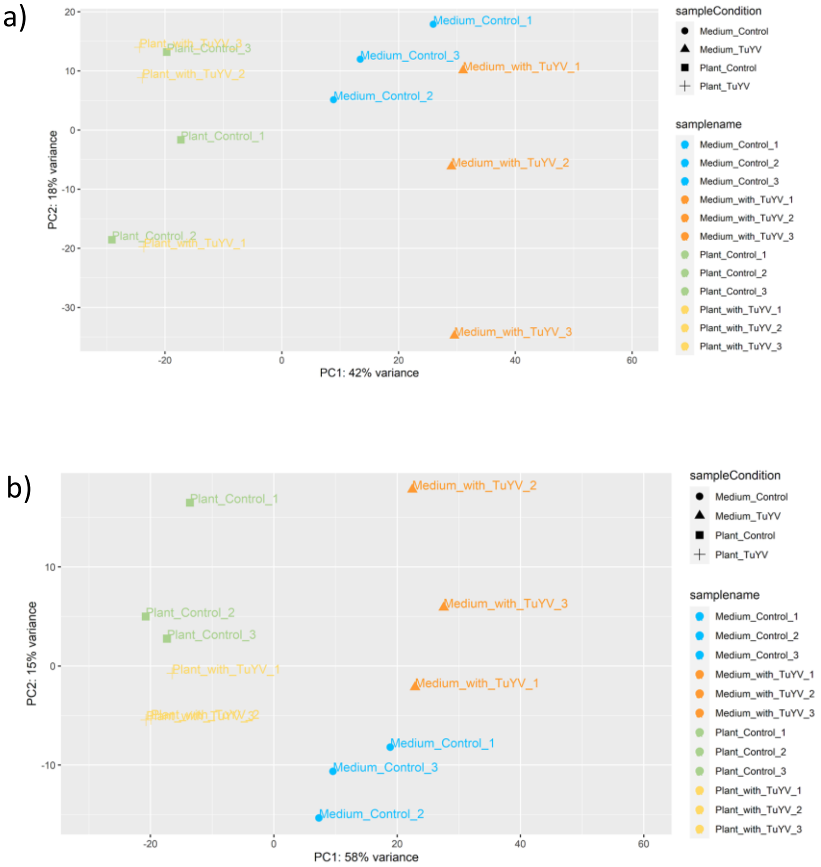

Additional file 5: Principal component analysis (PCA) of the RNA-Seq data showing samples distribution (NOISeq analysis) before (a) and (b) after batch correction. See Additional file 6 for raw data.
