# Supplementary material for: Differential gene expression in aphids following virus acquisition from plants or from an artificial medium
Source: BMC Genomics. 2022 Apr 30;23:333. doi: 10.1186/s12864-022-08545-1 (PMC9055738; doi:10.1186/s12864-022-08545-1)
Supplement: Supplementary file 7 — Additional file 7. Significant Gene Ontology (GO) categories of (a) molecular function (MF), (b) cellular components (CC) among the deregulated genes identified by the NOISeq analysis in the aphid M. persicae following feeding on TuYV-infected or non-infected plants. The percentage of deregulated genes from the total number of genes included in each GO category is indicated on the horizontal axis (% DE); counts: number of genes differentially expressed in the GO term. GO term boxed in blue represents categories potentially implicated in virus uptake and intracellular transport and GO term boxed in red categories potentially involved in aphid behavior and signal perception. [file 12864_2022_8545_MOESM7_ESM.pdf]

Additional file 7

a)

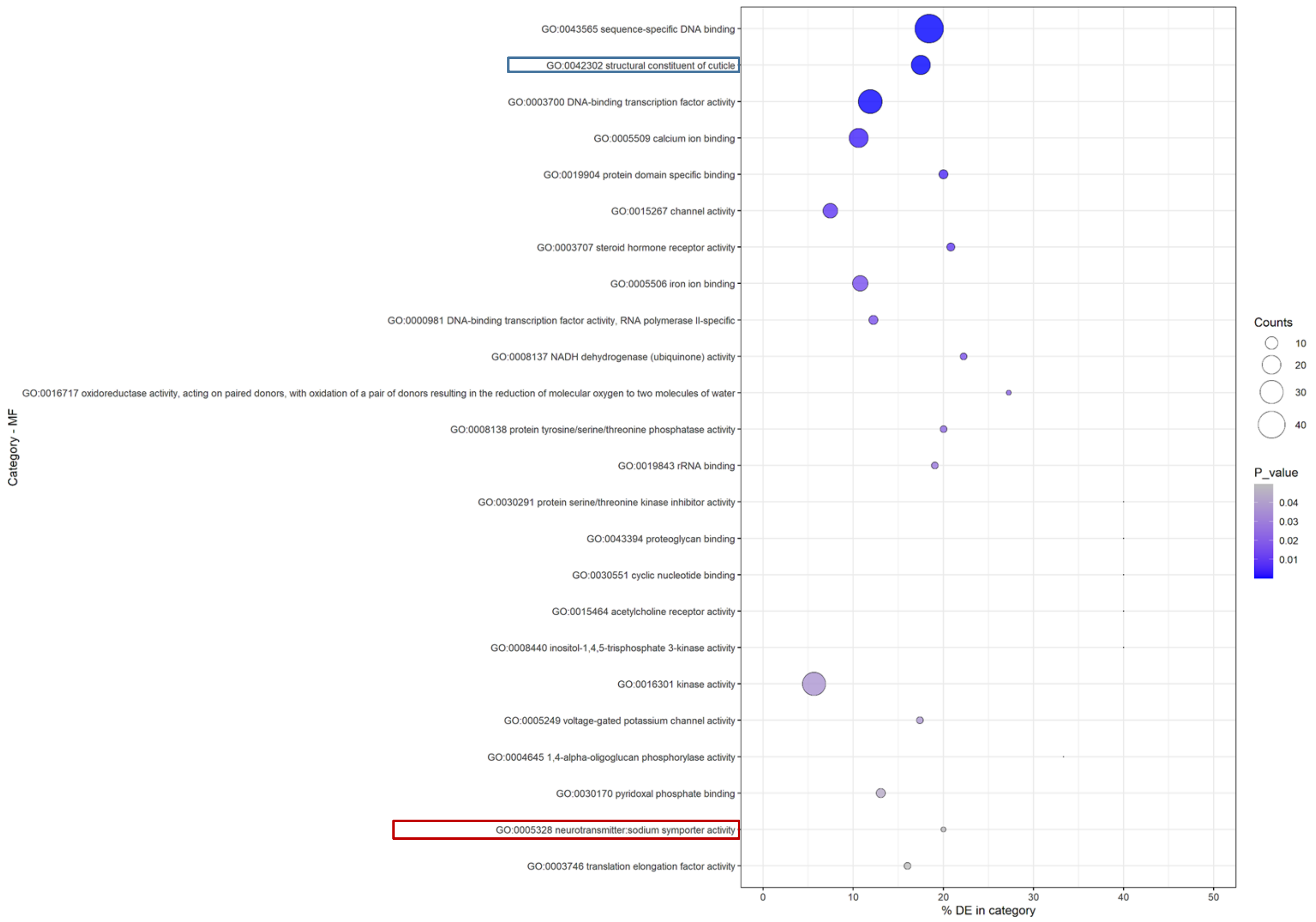

Additional file 7

b)

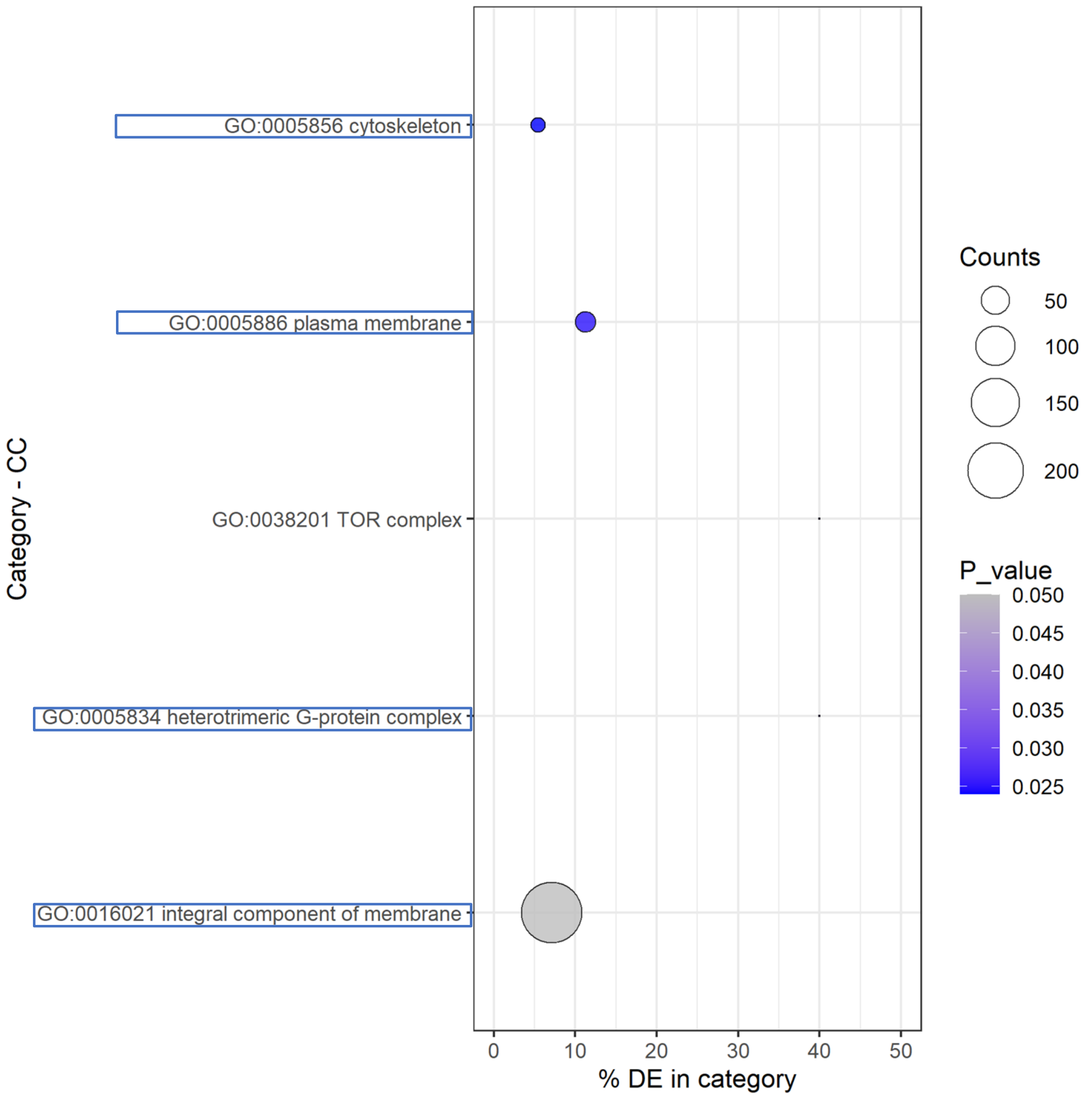

Additional file 7: Significant Gene Ontology (GO) categories of (a) molecular function (MF), (b) cellular components (CC) among the deregulated genes identified by the NOISeq analysis in the aphid *M. persicae* following feeding on TuYV-infected or non-infected plants. The percentage of deregulated genes from the total number of genes included in each GO category is indicated on the horizontal axis (% DE); counts: number of genes differentially expressed in the GO term. GO term boxed in blue represents categories potentially implicated in virus uptake and intracellular transport and GO term boxed in red categories potentially involved in aphid behavior and signal perception.
