# Supplementary material for: Differential gene expression in aphids following virus acquisition from plants or from an artificial medium
Source: BMC Genomics. 2022 Apr 30;23:333. doi: 10.1186/s12864-022-08545-1 (PMC9055738; doi:10.1186/s12864-022-08545-1)

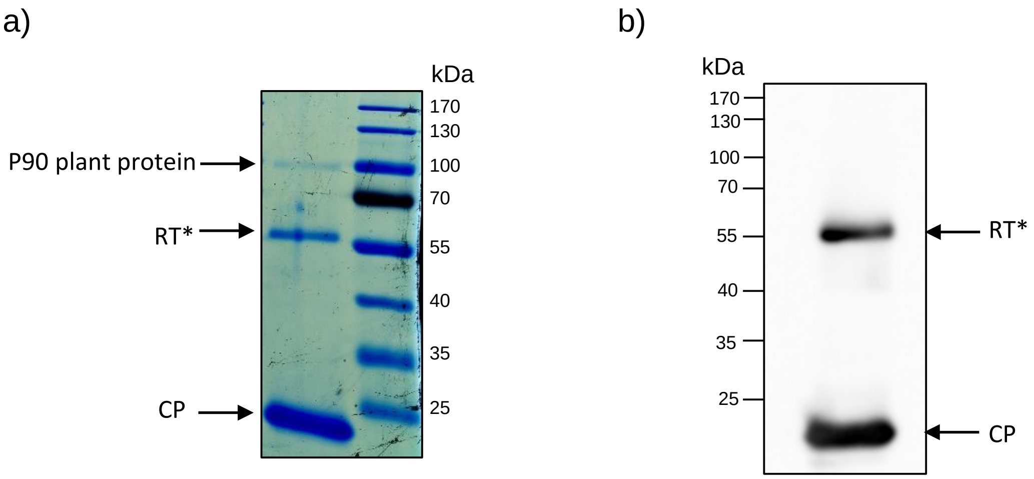

Additional file 8: Protein content of the purified extract prepared from TuYV-infected *M. perfoliata* and used to feed aphids artificially before RNASeq analysis. (a) Polyacrylamide gel electrophoresis of the TuYV purified extract (3 µg) stained with Coomassie blue; (b) Western blot analysis of the same purified extract. The blot was incubated with a mixture of antisera specific for the CP and RT*. CP: major coat protein (CP); RT*: minor coat protein (RT*). The band corresponding to a major plant protein (P90) in the TuYV purified preparation is indicated. The molecular mass markers are indicated in kDa.

Original blots

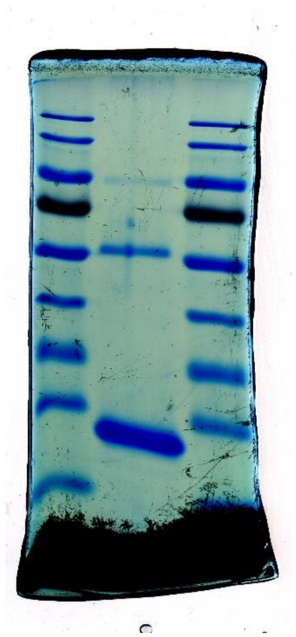

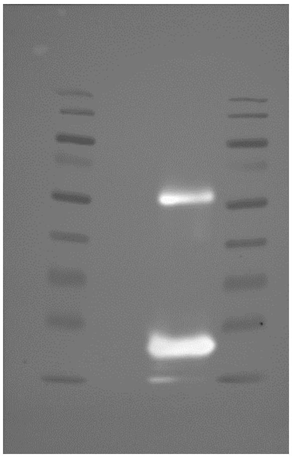

Supplement: Supplementary file 8 — Additional file 8. Protein content of the purified extract prepared from TuYV-infected M. perfoliata and used to feed aphids artificially before RNASeq analysis. (a) Polyacrylamide gel electrophoresis of the TuYV purified extract (3 µg) stained with Coomassie blue; (b) Western blot analysis of the same purified extract. The blot was incubated with a mixture of antisera specific for the CP and RT*. CP: major coat protein (CP); RT*: minor coat protein (RT*). The band corresponding to a major plant protein (P90) in the TuYV purified preparation is indicated. The molecular mass markers are indicated in kDa. [file 12864_2022_8545_MOESM8_ESM.pdf]
