# Supplementary material for: Differential gene expression in aphids following virus acquisition from plants or from an artificial medium
Source: BMC Genomics. 2022 Apr 30;23:333. doi: 10.1186/s12864-022-08545-1 (PMC9055738; doi:10.1186/s12864-022-08545-1)
Supplement: Supplementary file 10 — Additional file 10. Significant Gene Ontology (GO) categories of (a) biological process (BP), (b) molecular function (MF), (c) cellular components (CC) among the deregulated genes identified by the NOISeq analysis in the aphid M. persicae following feeding on artificial medium containing TuYV purified particles or artificial medium only. The percentage of deregulated genes from the total number of genes included in each GO category is indicated on the horizontal axis (% DE); counts: number of genes differentially expressed in the GO term. GO term boxed in blue represents categories potentially implicated in virus uptake and intracellular transport. [file 12864_2022_8545_MOESM10_ESM.pdf]

Additional file 10

a)

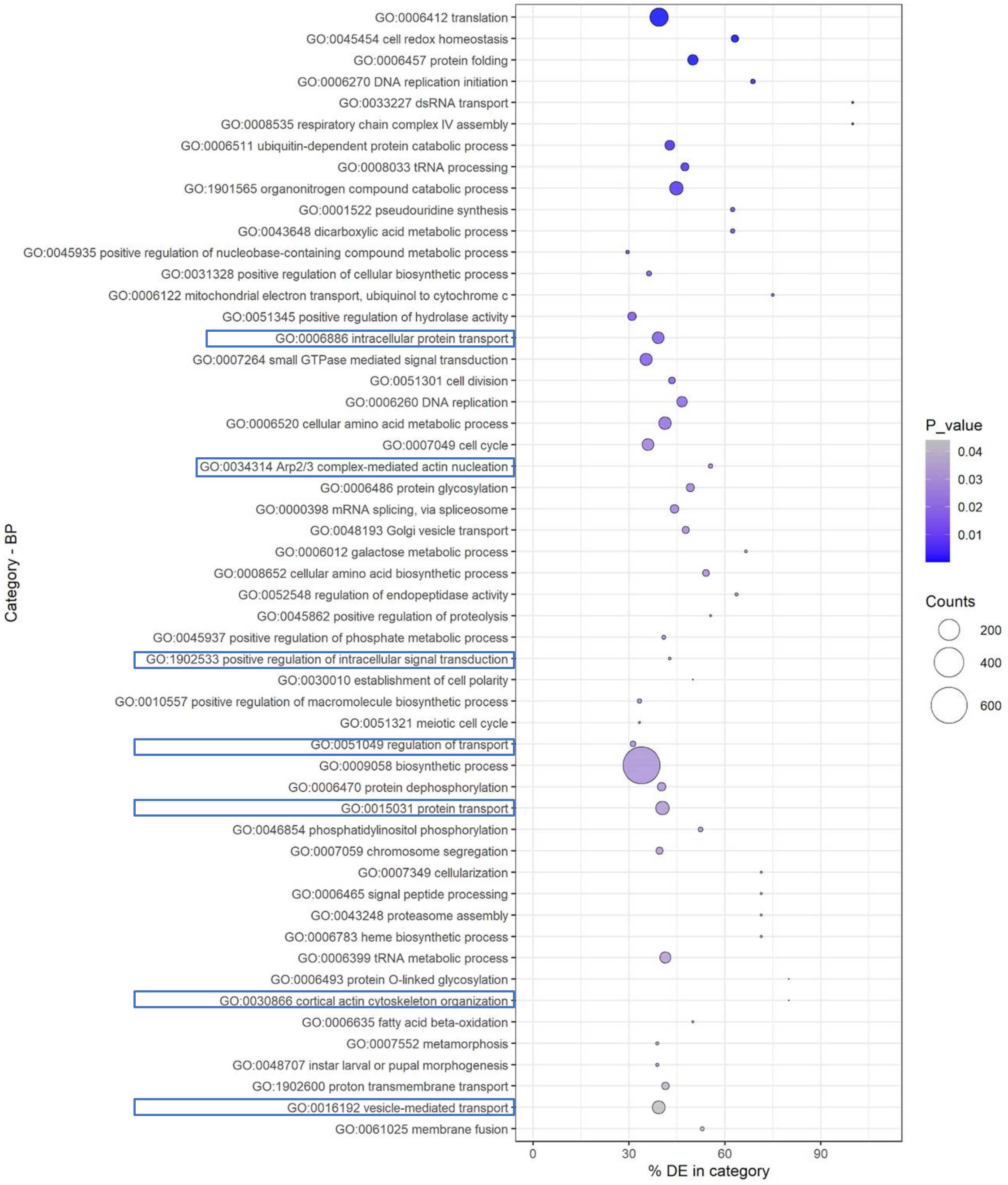

Additional file 10

b)

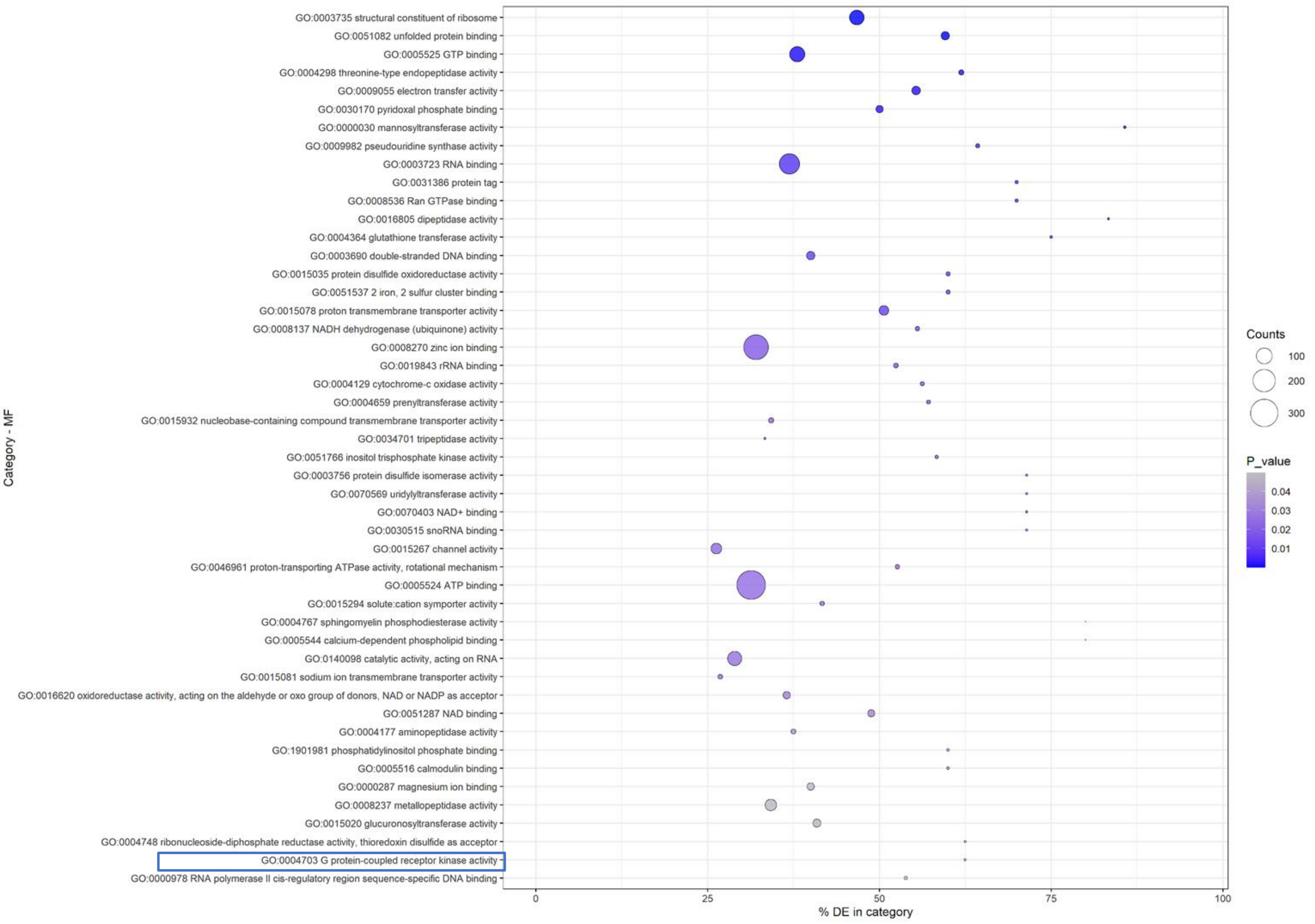

Additional file 10

c)

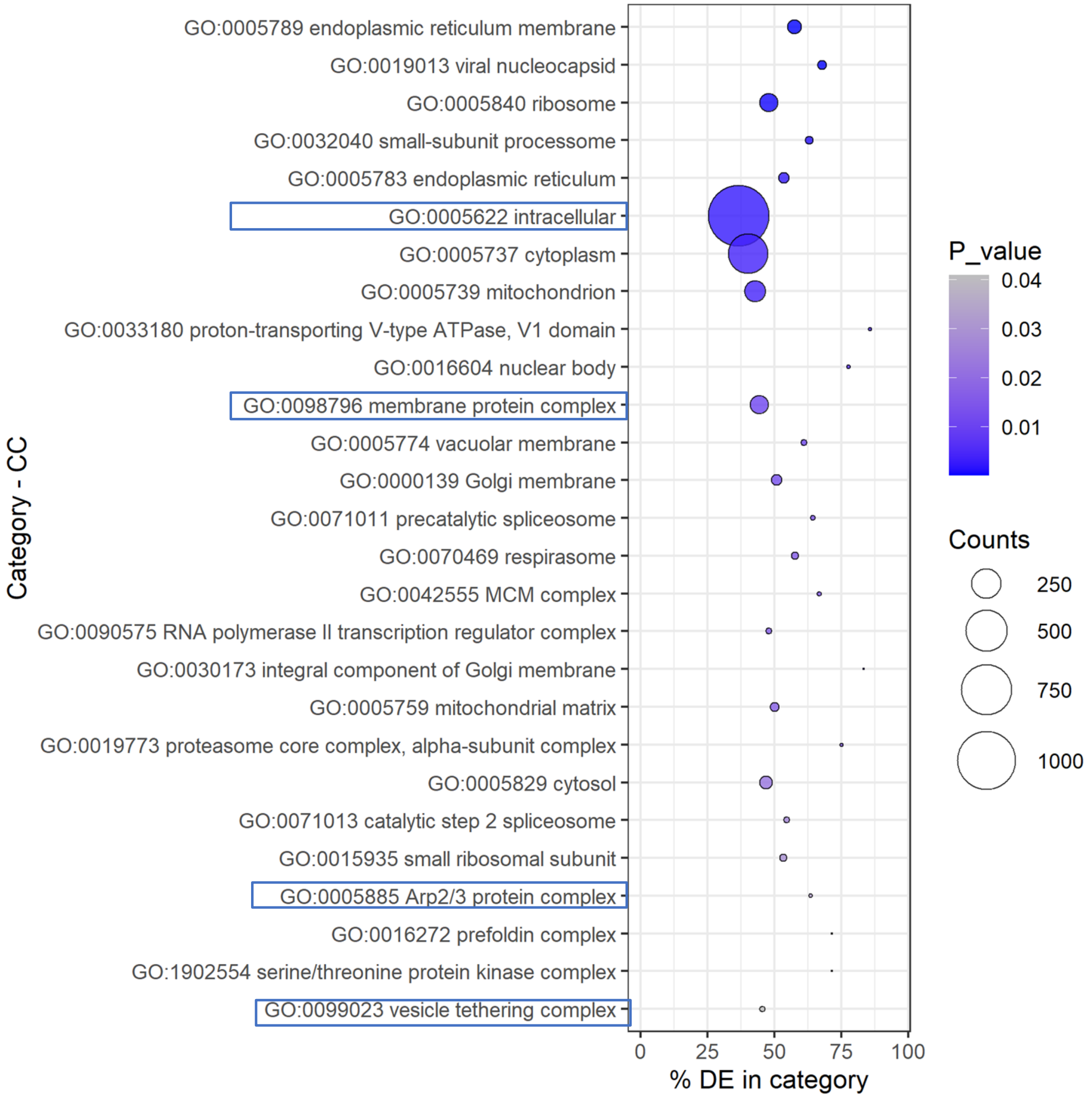

Additional file 10: Significant Gene Ontology (GO) categories of (a) biological process (BP), (b) molecular function (MF), (c) cellular components (CC) among the deregulated genes identified by the NOISeq analysis in the aphid *M. persicae* following feeding on artificial medium containing TuYV purified particles or artificial medium only. The percentage of deregulated genes from the total number of genes included in each GO category is indicated on the horizontal axis (% DE); counts: number of genes differentially expressed in the GO term. GO term boxed in blue represents categories potentially implicated in virus uptake and intracellular transport.
