# Supplementary material for: Differential gene expression in aphids following virus acquisition from plants or from an artificial medium
Source: BMC Genomics. 2022 Apr 30;23:333. doi: 10.1186/s12864-022-08545-1 (PMC9055738; doi:10.1186/s12864-022-08545-1)
Supplement: Supplementary file 11 — Additional file 11. Common deregulated genes in aphids between plant and artificial medium feeding conditions with the DESeq2 program. [file 12864_2022_8545_MOESM11_ESM.pdf]

Additional file 11 : Common deregulated genes in aphids between plant and artificial medium feeding conditions with the DESeq2 program

Gene	annotation	log2FC<br>On artificial medium	P-value<br>On artificial medium	log2FC<br>On plants	P-value<br>On plants
MYZPE13164_G006_v1.0_000159200	Juvenile hormone-inducible protein 26	-0.57	3.10E-02	0.65	7.85E-03
MYZPE13164_G006_v1.0_000137120	Nuclease harbi1-like protein	-1.46	2.68E-02	1.35	3.10E-02
MYZPE13164_G006_v1.0_000166250	Unknown protein	1.26	2.07E-04	-0.65	1.77E-02
MYZPE13164_G006_v1.0_000166240	Unknown protein	1.30	3.60E-03	-1.02	9.27E-04
