# Supplementary material for: Differential gene expression in aphids following virus acquisition from plants or from an artificial medium
Source: BMC Genomics. 2022 Apr 30;23:333. doi: 10.1186/s12864-022-08545-1 (PMC9055738; doi:10.1186/s12864-022-08545-1)
Supplement: Supplementary file 12 — Additional file 12. Locomotor activity of viruliferous and non-viruliferous M. persicae fed on plants or on artificial medium. (a) maximum zone reached and (b) duration (in seconds) spent in each zone. Box plot show median (line), 25-75% percentiles (box) and 10-90% percentiles (whisker). Letters indicate significant differences between aphid status with the GLM followed by multiple comparisons; p-value<0,05. [file 12864_2022_8545_MOESM12_ESM.pdf]

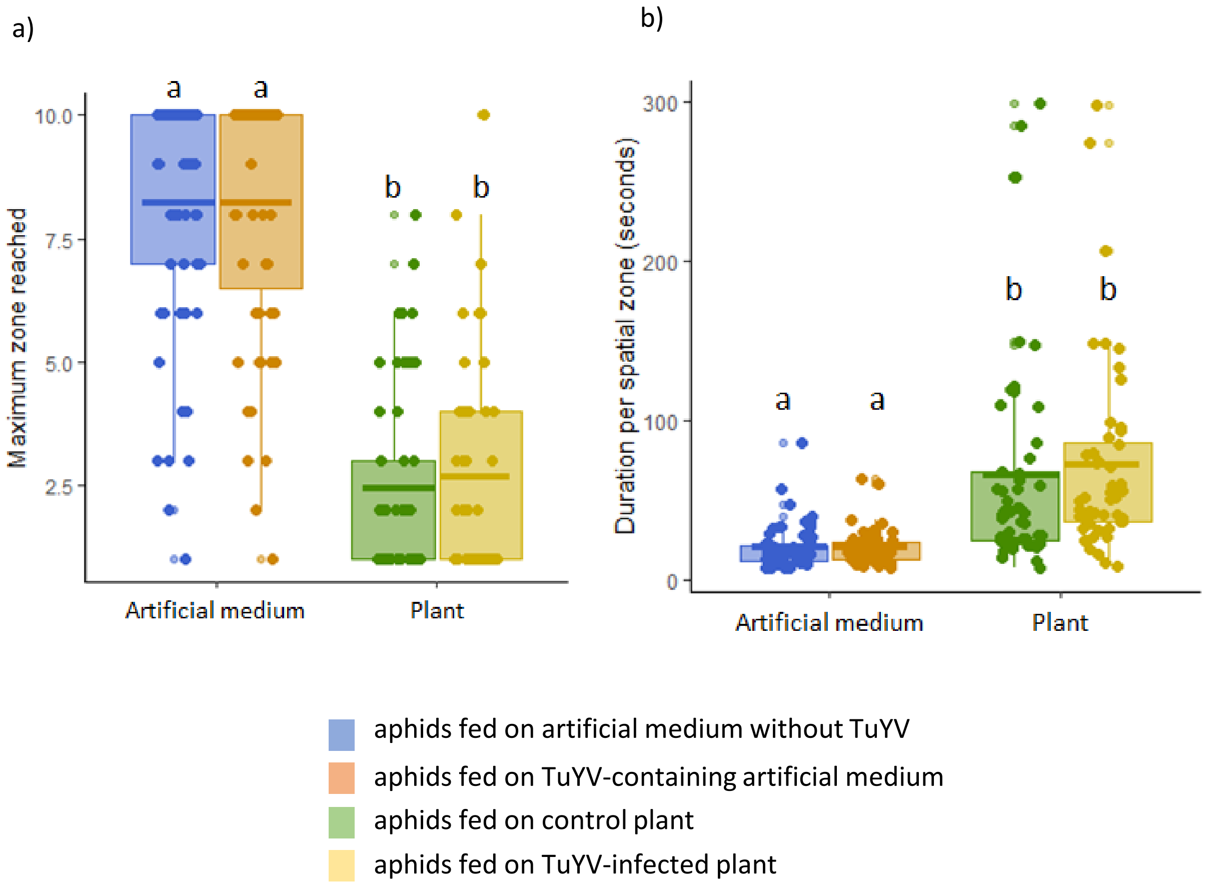

Additional file 12: Locomotor activity of viruliferous and non-viruliferous *M. persicae* fed on plants or on artificial medium. (a) maximum zone reached and (b) duration (in seconds) spent in each zone. Box plot show median (line), 25-75% percentiles (box) and 10-90% percentiles (whisker). Letters indicate significant differences between aphid status with the GLM followed by multiple comparisons; p-value<0,05.
